# Association between Breastfeeding and Restrictive Spirometric Pattern in Women Aged over 40 Years: A Cross-Sectional Study

**DOI:** 10.3390/ijerph192316291

**Published:** 2022-12-05

**Authors:** Hyeokjoo Jang, Sebin Kwon, Bumyeol Lee, Gahyeon Kim, Wonjeong Chae, Sung-In Jang

**Affiliations:** 1 College of Medicine, Yonsei University, Seoul 03722, Republic of Korea; 2 Institute of Health Services Research, Yonsei University, Seoul 03722, Republic of Korea; 3 Department of Health Policy and Management, Graduate School of Public Health, Yonsei University, Seoul 03722, Republic of Korea; 4 Department of Preventive Medicine, Yonsei University, Seoul 03722, Republic of Korea

**Keywords:** breastfeeding, pulmonary function, restrictive lung disease, restrictive spirometric pattern, parous women

## Abstract

Objectives: Restrictive spirometric pattern (RSP) has a prevalence of 5.4–9.2% and is associated with various respiratory symptoms, comorbidities, and increased mortality. Breastfeeding has important effects on maternal health; however, the effects of breastfeeding on pulmonary function remain unclear. This study aimed to investigate the effects of breastfeeding on maternal pulmonary function, particularly the risk of RSP. Methods: Retrospective, cross-sectional observational study enrolling parous women aged >40 years who participated in the Korea National Health and Nutrition Examination Survey from 2013–2018. RSP was defined using the FEV1/FVC ratio and FVC outcomes of the pulmonary function test. The adjusted odds ratios (OR) for RSP were calculated using multivariate logistic regression. Results: Of 9261 parous women, 913 (9.9%) had RSP. Breastfeeding (≥1 month) was associated with a reduced risk of RSP (OR: 0.75 [0.60–0.92]) when adjusted for age, body mass index, smoking status, other diseases, socioeconomic status, and maternal risk factors. The adjusted ORs for RSP for women decreased further with increasing duration of breastfeeding (*p* for trend: 0.0004). The FEV1, FVC, and FVC% were higher in women who breastfed than in those who did not breastfeed (by 0.0390 L, 0.0521 L, 0.9540% *p*, respectively). Conclusions: There is an association between breastfeeding and pulmonary function in parous women. Breastfeeding was associated with a lower prevalence of RSP in parous women aged >40 years old, suggesting that breastfeeding may have a beneficial effect on maternal pulmonary function.

## 1. Introduction

Most diseases of the respiratory system are classified into three categories according to their patterns: restrictive lung diseases, obstructive lung diseases, and vascular diseases [1,2,3]. Restrictive lung disease is characterized by a decrease in total lung volume due to restricted lung expansion. This causes the patients’ breathing to become more difficult, leading to inefficient ventilation and oxygenation [2,4]. Restrictive lung disease can be classified into three types depending on its pathophysiology: parenchymal disease, neuromuscular weakness, and chest wall/pleural diseases [5]. Each heterogeneous set of diseases includes hundreds of specific diagnoses [1,2,3,4,5].

Restrictive lung disease can be diagnosed with a low total lung capacity (TLC) and a normal FEV1/FVC ratio. The threshold values for TLC and FEV1/FVC ratios are 80% of the reference value and 0.7, respectively [6,7]. However, TLC measurement is rarely used in clinical practice to diagnose restrictive lung disease due to the technical limitations of spirometry. Instead, a restrictive spirometric pattern (RSP), determined by FEV1/FVC ratio ≥ 70% and FVC% < 80%, is often used [6,7]. RSP is common in the general population, with a prevalence ranging from 5.4% to 9.2% in data from the US National Health and Nutrition Examination Survey (NHANES) [7,8]. Recently, RSP has been reported to be associated with an increased incidence of respiratory symptom burden [9,10], functional limitations, such as higher mMRC dyspnea scores [11], comorbidities (such as metabolic syndrome and diabetes mellitus [12,13]), and adverse outcomes, including increased mortality [7,9].

Breastfeeding is a major health concern worldwide. Previous studies have shown that breastfeeding is beneficial for both mothers and children [14,15]. In particular, breastfeeding has recently been shown to reduce the risk of chronic diseases such as cardiovascular disorders, including hypertension, type II diabetes mellitus, metabolic syndrome, NAFLD, and ovarian cancer in parous women [16,17]. However, to the best of our knowledge, no study has investigated the relationship of breastfeeding with RSP or pulmonary function in parous women. Therefore, this study aimed to identify the effects of breastfeeding on maternal pulmonary function, especially the risk of RSP, in women aged >40 years using representative nationwide survey data. Furthermore, this study investigated whether the duration of breastfeeding was related to the risk of RSP.

## 2. Methods

### 2.1. Data Source, Study Design, and Population

The Korean NHANES (KNHANES) is a nationwide cross-sectional survey conducted by the Korea Disease Control and Prevention Agency (KCDA) to assess the health and nutritional status of the Korean population [18].

**Figure 1:**
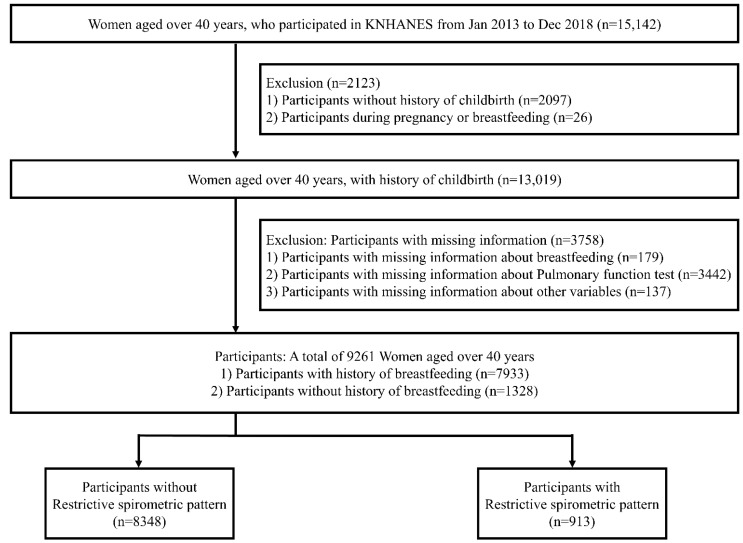
Flow chart of study population selection.

We collected data from women aged over 40 years who participated in the KNHANES from January 2013 to December 2018 (n = 15,142). We excluded participants with no history of childbirth (n = 2097), during pregnancy or breastfeeding (n = 26), with missing information about breastfeeding (n = 179), pulmonary function test (PFT, n = 3442), or regarding other variables (n = 137). Finally, 9261 women aged over 40 years with a history of childbirth were analyzed (Figure 1).

### 2.2. Study’s Main Variables

A restrictive spirometric pattern (RSP) was defined as a pre-bronchodilator FEV1/FVC ≥ 70% and FVC < 80% using the pulmonary function test, according to the ATS criteria (fixed-ratio criteria) [19]. Information on breastfeeding was extracted from the KNHANES survey. Experienced researchers investigated the history and total duration of breastfeeding through interviews. Based on the survey question, “Have you ever breastfed for more than 1 month?” those who answered “no” were defined as the non-breastfeeding group. For those in the breastfeeding group, the breastfeeding period was evaluated for at least one month of breastfeeding. The duration of breastfeeding was then categorized into 1–6 months, 7–12 months, 13–18 months, 19–24 months, and more than 24 months.

### 2.3. Covariates and Measurements

We extracted the following data from the KNHANES database for the analyses: duration of breastfeeding; FEV1, FVC, and FVC% in PFTs; RSP; COPD; age; height; body weight; smoking status; history of asthma, pulmonary tuberculosis, hypertension, and diabetes mellitus; region; employment status; education level; household income level; number of pregnancies; number of children breastfed; age at menarche; age at first delivery; and age at the last delivery.

The body mass index (BMI) was calculated as body weight per square of height (kg/m^2^), and participants were categorized into underweight (<18.5 kg/m^2^), normal (≥18.5 to <25 kg/m^2^), and obese (≥25 kg/m^2^) according to BMI values. Smoking status was classified as ever smoker, former smoker, or never smoker. An ever smoker refers to a person who smoked more than 100 cigarettes during their lifetime, and a former smoker is a person who smoked less than 100 cigarettes during their lifetime and now does not smoke. Never smoked was defined as an individual who had never smoked in their life. The region was categorized into capital (including Seoul, Incheon, and Gyeonggi-do) and non-capital regions. Employment status was classified into three categories: blue-collar (labor type workers), white-collar (administrative, managerial type workers), and unemployed workers. The educational level was categorized into four categories according to the highest level of education: elementary or lower, middle, high or secondary, and college or higher. Household income levels were categorized into quartiles: very low, low, high, and very high. Spirometry (PFT) was performed to measure the FEV1, FVC, and FVC%. Dry rolling seal spirometers, which were used until June 2016, were replaced with vyntus spiro in July 2016.

### 2.4. Statistical Analysis

All statistical analyses were performed using SAS software (version 9.4; SAS Institute, Cary, NC, USA). Categorical variables are expressed as numbers and proportions (%), and continuous variables are expressed as medians (interquartile ranges).

Differences in variables between participants with and without RSP and differences between participants who had breastfed and those who did not were evaluated using chi-square tests. The association between breastfeeding and RSP was calculated using multivariate logistic regression, which was adjusted for age, smoking status, asthma, pulmonary tuberculosis, hypertension, diabetes mellitus (diagnosed vs. never diagnosed), region of residence, employment, education level, house income level, parity, age at menarche, age at the first delivery, age at the last delivery, and examined year.

To assess whether a linear relationship existed between each categorical variable and RSP, it was defined as a continuous variable, and multiple logistic regression was performed (*p* for trend). The association between breastfeeding duration in six categories and RSP was also tested by multivariate logistic regression adjusted for the same covariables as presented above. The generalized linear method was used to determine the relationship between breastfeeding and secondary outcomes, including FEV1 (L), FVC (L), FVC percentage (%), and FEV1/FVC ratio, and a generalized linear method was used.

Finally, pre-specified subgroup analyses were performed to assess the consistency of the association between breastfeeding and RSP among various subgroups. Subgroups were defined using the same covariables used in multiple logistic regression, and interaction tests were used to determine the potential interaction effect between breastfeeding and the covariables (*p* for interaction). All variables with a *p*-value < 0.05 were considered statistically significant.

## 3. Results

### 3.1. Demographic Characteristics of the Participants

The demographic characteristics of the participants are summarized in Table 1 and Supplementary Table S1. A total of 9261 participants were included in this study. Among them, 913 (9.9%) had RSP, and 1328 (14.3%) did not breastfeed. The mean (SD) values of participants were 57.5 (10.7) years for age, 24.0 (3.2) kg/m^2^ for BMI, 2.30 (0.45) L for FEV1, 2.91 (0.51) L for FVC, 92.76% (11.58%) for FVC%, and 0.79 (0.06) for FEV1/FVC ratio, respectively. Compared with the non-RSP group, the RSP group had a higher mean age (61.7 vs. 57.0 years), BMI (25.4 vs. 23.9 kg/m^2^), and age at menarche (14.8 vs. 14.5 years), and lower age at the first delivery (24.4 vs. 25.1 years) and at the last delivery (29.2 vs. 29.5 years).

### 3.2. Association between RSP and Breastfeeding

The result of logistic regression analysis on the association between RSP and the breastfeeding group showed a lower adjusted odds ratio (OR) for RSP among the breastfeeding group (OR: 0.75 [0.60–0.92], *p* = 0.007; Table 2). By classifying the duration of breastfeeding, adjusted ORs for RSP in participants with breastfeeding durations of 1–6 months, 7–12 months, 13–18 months, 19–24 months, and more than 24 months compared with the non-breastfeeding group were 0.86 [0.65–1.14], 0.79 [0.59–1.06], 0.82 [0.60–1.14], 0.74 [0.57–0.96], and 0.63 [0.49–0.81], respectively. The *p*-value for the trend according to breastfeeding was 0.0004.

For other independent variables, underweight and obese participants had higher ORs for RSP than those of normal participants. (OR: 1.76 [1.08–2.85], 2.00 [1.73–2.31], respectively); and participants with hypertension and diabetes mellitus had higher ORs for RSP compared with participants without hypertension and diabetes mellitus. (OR: 1.19 [1.01–1.40], 1.46 [1.19–1.79], respectively)

### 3.3. Correlation between Breastfeeding and the Results of the Respiratory Function Test

In reference to the non-breastfeeding group, the breastfeeding group had a higher FEV1 (by 0.0390 L, *p* = 0.0001), FVC (by 0.0521 L, *p* < 0.0001), and FVC percentage (by 0.9540% *p*, *p* = 0.0051). The FEV1/FVC ratio showed no statistically significant difference (*p* = 0.1956). The *p*-values for the trend by the duration of breastfeeding were 0.0004 for FEV1, <0.0001 for FVC, 0.0002 for FVC %, and 0.1956 for the FEV1/FVC ratio (Table 3).

### 3.4. Subgroup Analyses

**Figure 2:**
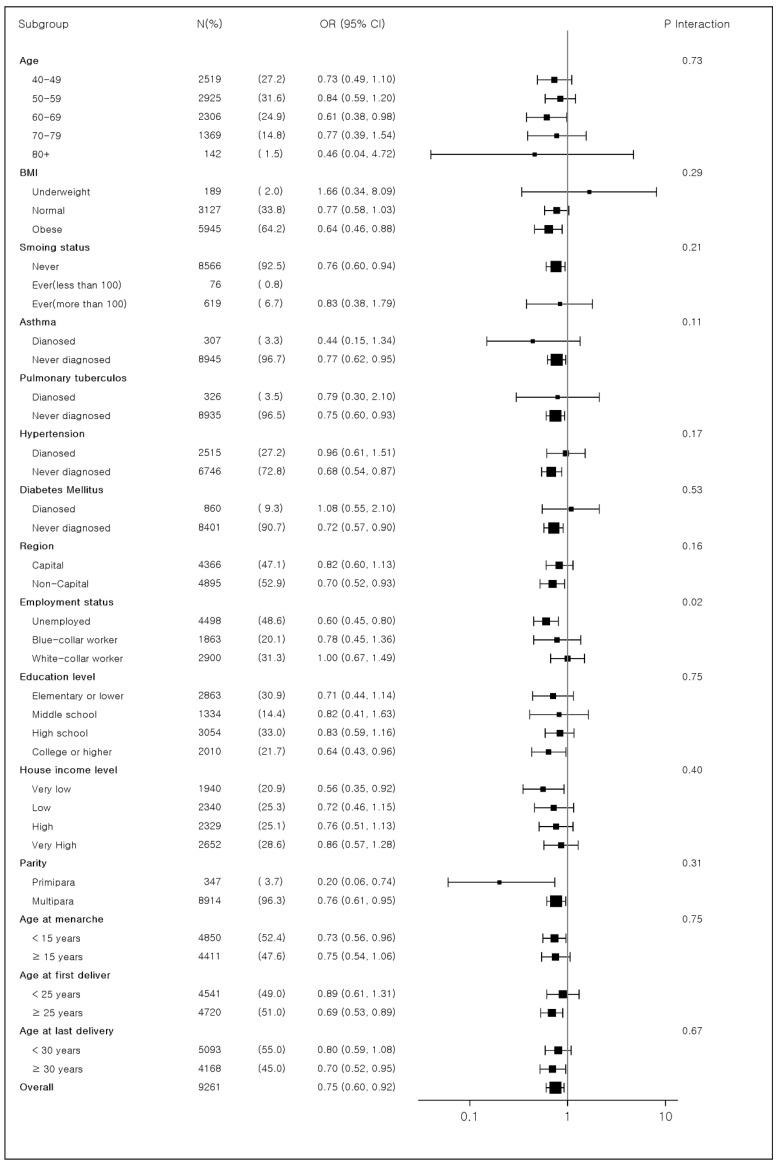
Forest plot of subgroup analysis of the association between breastfeeding and restrictive spirometric pattern stratified by covariates.

Figure 2 shows a forest plot of subgroup analyses. In pre-specified subgroup analyses, subgroups defined by employment status (unemployed vs. white-collar worker vs. blue-collar worker) showed statistically significant interactions with breastfeeding years (*p* = 0.0218).

Among the three subpopulations, the OR of having RSP in the breastfeeding group compared with the non-breastfeeding group was lowest in the subpopulation who were unemployed (OR: 0.60 [0.45–0.80]), middle in the subpopulation of blue-collar workers (OR: 0.78 [0.45–1.36]), and highest in the subpopulation of white-collar workers (OR: 1.00 [0.67–1.49]). However, the subgroups defined by other variables did not show significant interaction effects with breastfeeding.

## 4. Discussion

In this study, we demonstrated a negative correlation between breastfeeding and RSP in parous women, despite adjusting for all possible confounder variables. According to our main analysis, the risk of RSP in women with a history of breastfeeding was approximately 25% lower than in those with no history of breastfeeding. (OR: 0.75 [0.60–0.92], *p*:0.007) This protective effect of breastfeeding against RSP was also consistently observed in most of the subgroups. The subpopulation diagnosed with diabetes mellitus (OR: 1.08 [0.55–2.10]) and those with a lower BMI (OR: 1.66 [0.34–8.09]) were the only exceptions; however, the relationship between breastfeeding and RSP was not statistically significant in these two subgroups. Additionally, the adjusted OR decreased further with increasing the duration of breastfeeding. (*p* for trend: 0.0004) The risk of RSP in women who breastfed for 19–24 months and more than 24 months was significantly lower compared with the non-breastfeeding group (OR: 0.74 [0.57–0.96], 0.63 [0.49–0.81], respectively), while women who did for 1–6 months, 7–12 months, 13–18 months were not (0.86 [0.65–1.14], 0.79 [0.59–1.06], 0.82 [0.60–1.14]). This suggests that the protective effect of breastfeeding against RSP may be strengthened by increasing the duration of breastfeeding. Other factors independently associated with an increased risk of RSP were age, BMI, doctor-diagnosed hypertension, and doctor-diagnosed diabetes mellitus. These risk factors have already been identified in previous studies based on KHANES and US NHANES. [12,13,20]

Many recent studies have shown the health effects of breastfeeding on mothers. As breastfeeding suppresses gonadotropins, breastfeeding probably has protective effects against ovarian cancer. [17] Additionally, breastfeeding activates central neuroendocrine pathways, including oxytocin and prolactin, and lactation itself positively affects glucose and insulin homeostasis. These findings may explain the protective effects of breastfeeding against hypertension and type 2 diabetes mellitus. Breastfeeding has also been reported to be associated with a lower incidence of other diseases, including metabolic syndrome, obesity [16,17], Alzheimer’s disease [21], gall bladder disease [22], rheumatoid arthritis [23], hip fractures, and osteoporosis [24]. However, before this study, the association between breastfeeding and pulmonary function had not been investigated.

Since spirometry cannot measure TLC, restrictive lung disease cannot be diagnosed by spirometry alone, whereas RSP can be defined by FEV1 and FVC. Although RSP does not reflect the actual lung volume, studies have reported that it is also meaningful [25]. First, RSP is associated with a higher burden of chronic respiratory symptoms and functional limitations [9,10,11]. According to Soriano et al., the patient group with RSP showed more phlegm, dyspnea, and wheezing than the normal group and reported a significant worsening of the mMRC dyspnea score, which is comparable to the COPD group [10]. Second, RSP is related to comorbidities, such as obesity, metabolic syndrome, and diabetes mellitus [12,13]. Third, RSP is associated with adverse outcomes, such as lung cancer, cardiovascular disease, and increased mortality. According to a large study in Sweden, RSP is an independent predictor of lung cancer, especially squamous cell carcinoma and small cell carcinoma, but not adenocarcinoma [26]. Finally, it has been reported that RSP is associated with increased mortality [7,9].

Although the biological mechanisms underlying the protective effects of breastfeeding against RSP are unclear, one possible key mechanism that could explain the relationship between the two is systemic inflammation. Mannino et al. showed that the presence of RSP was associated with higher levels of systemic CRP and fibrinogen and that the levels of markers were comparable with those of moderate COPD [6]. Additionally, previous studies have shown that systemic inflammation is associated with impaired lung function, especially lower FVC. Several studies have shown that decreased FVC is associated with higher levels of CRP [27], fibrinogen [28], and other inflammation-sensitive plasma proteins (haptoglobin, ceruloplasmin, α1-antitrypsin, and orosomucoid) [29]. According to a prospective cohort study conducted by Ahn et al., the level of the pro-inflammatory cytokine IL-6 at 6 months postpartum was lower in women who primarily practiced breastfeeding than in women who practiced bottle feeding [30]. Groer et al. also showed that exclusively breastfeeding mothers were more likely to have lower IFN-γ levels and IFN-γ/IL-10 ratios at weeks 4 to 6 postpartum than exclusively formula-feeding mothers [31]. Together, these findings suggest that systemic inflammation may explain the link between breastfeeding and a lower prevalence of RSP. However, further studies are needed to identify the association between systemic inflammation and breastfeeding and whether the anti-inflammatory effects of breastfeeding last until the later period of life. Furthermore, systemic inflammation may not be the only explanation for the lower prevalence of RSP in breastfeeding mothers. For instance, breastfeeding may affect factors involved in the pathogenesis of restrictive lung disease. Metabolic factors known to be related to breastfeeding and restrictive lung disease may also play a role. Therefore, further studies are required to identify the mechanisms underlying the protective effects of breastfeeding against RSP.

This study has several strengths. This was the first study to determine the association between breastfeeding and maternal pulmonary function, particularly the prevalence of RSP. We hope that this study serves as a meaningful first step in investigating the relationship between breastfeeding and maternal pulmonary function. Second, we used data from the KNHANES data, which is sufficiently large to represent the entire Korean population. Third, the effects of any known risk factors for maternal RSP or potentially confounding factors were corrected using multivariate logistic regression.

However, this study also has some limitations. First, this was a cross-sectional study that is not suitable for evaluating the causal effect of breastfeeding on RSP, despite the significant association between breastfeeding and the prevalence of RSP. Second, we used RSP instead of restrictive lung disease due to the lack of information about TLC. Third, a standard definition of RSP has not yet been established. RSP is defined in two ways: by the fixed ratio criterion and by the lower limit normal (LLN) criterion [25,32]. We used a fixed ratio criterion instead of the LLN criterion to define RSP, although using a fixed ratio criterion can lead to overdiagnosis of obstructive lung disease in older age groups [33]. Fourth, the data were collected in the form of a survey, which could have caused recall bias. However, it has been reported that information about the breastfeeding of the respondent can be precisely recalled [34]. The KHANES survey data contains general health data in a large population that does not include viral or bacterial infection history, which we could not include in the study. Nevertheless, we used a diagnosis history of asthma and pulmonary tuberculosis to potential factors related to spirometry; we suggest further study using data with detailed health information.

## 5. Conclusions

In conclusion, this study showed that breastfeeding is associated with a reduced prevalence of RSP, which means that breastfeeding can have beneficial effects on maternal lung function. Further studies should be conducted to evaluate restrictive lung disease in terms of TLC and focus on causal effects or pathophysiology.

## Figures and Tables

**Table 1:** Demographic characteristics of participants according to restrictive spirometric pattern.

Variables	Total (n)		RSP		Non-RSP		*p*-Value
	n	%	n	%	n	%	
	9261		913	9.9	8348	90.1	
**Breastfeeding duration (months)**							0.0006
None	1328	14.3	128	9.6	1200	90.4	
Yes	7933	85.7	785	9.9	7148	90.1	
1–6	1247	13.5	96	7.7	1151	92.3	
7–12	1051	11.3	87	8.3	964	91.7	
13–18	780	8.4	65	8.3	715	91.7	
19–24	1487	16.1	150	10.1	1337	89.9	
25-	3368	36.4	387	11.5	2981	88.5	
**Age (years)**	57.5 (10.7) †		61.7 (10.4) †		57.0 (10.6) †		<0.0001
40–49	2519	27.2	126	5.0	2393	95.0	
50–59	2925	31.6	263	9.0	2662	91.0	
60–69	2306	24.9	269	11.7	2037	88.3	
70–79	1369	14.8	222	16.2	1147	83.8	
80+	142	1.5	33	23.2	109	76.8	
**BMI (kg/m^2^)**	24.0 (3.2) †		25.4 (3.8) †		23.9 (3.2) †		<0.0001
Underweight	189	2.0	20	10.6	169	89.4	
Obese	3127	33.8	466	14.9	2661	85.1	
Normal	5945	64.2	427	7.2	5518	92.8	
**FEV1 (L)**	2.30 (0.45) †		1.81 (0.30) †		2.36 (0.43) †		
**FVC (L)**	2.91 (0.51) †		2.27 (0.32) †		2.98 (0.48) †		
**FVC Percentage (%)**	92.76 (11.58) †		74.22 (5.30) †		94.79 (10.20) †		
**FEV1/FVC**	0.79 (0.06) †		0.80 (0.05) †		0.79 (0.06) †		
**Smoking status**							0.6915
Ever (less than 100)	76	0.8	8	10.5	68	89.5	
Ever (more than 100)	619	6.7	55	8.9	564	91.1	
Never	8566	92.5	850	9.9	7716	90.1	
**Asthma**							0.0580
Diagnosed	307	3.3	40	13.0	267	87.0	
Never diagnosed	8954	96.7	873	9.7	8081	80.3	
**Pulmonary tuberculosis**							0.0937
Diagnosed	326	3.5	41	12.6	285	87.4	
Never diagnosed	8935	96.5	872	09.8	8063	90.2	
**Hypertension**							<0.0001
Diagnosed	2515	27.2	368	14.6	2147	85.4	
Never diagnosed	6746	72.8	545	08.1	6201	91.9	
**Diabetes Mellitus**							<0.0001
Diagnosed	860	9.3	153	17.8	707	82.2	
Never diagnosed	8401	90.7	760	9.0	7641	91.0	
**Region**							0.1346
Capital	4366	47.1	409	9.4	3957	90.6	
Non-Capital	4895	52.9	504	10.3	4391	89.7	
**Employment status**							<0.0001
Blue-collar worker	1863	20.1	194	10.4	1669	89.6	
White-collar worker	2900	31.3	220	7.6	2680	92.4	
Unemployed	4498	48.6	499	11.1	3999	88.9	
**Education level**							<0.0001
Elementary or lower	2863	30.9	366	12.8	2497	87.2	
Middle school	1334	14.4	149	11.2	1185	88.8	
High school	3054	33.0	263	8.6	2791	91.4	
College or higher	2010	21.7	135	6.7	1875	93.3	
**House income level**							<0.0001
Very low	1940	20.9	253	13.0	1687	87.0	
Low	2340	25.3	233	10.0	2107	90.0	
High	2329	25.1	226	9.7	2103	90.3	
Very high	2652	28.6	201	7.6	2451	92.4	
**Parity**							0.0052
Primipara	347	3.7	19	5.5	328	94.5	
Multipara	8914	96.3	894	10.0	8020	90.0	
**Age at menarche**	14.6 (1.9) †		14.8 (2.0) †		14.5 (1.9) †		0.0010
<15 years	4850	52.4	431	8.9	4419	91.1	
≥15 years	4411	47.6	482	10.9	3929	89.1	
**Age at the first delivery**	25.0 (3.9) †		24.4 (3.8) †		25.1 (3.9) †		<0.0001
<25 years	4541	49.0	514	11.3	4027	88.7	
≥25 years	4720	51.0	399	8.5	4321	91.5	
**Age at the last delivery**	29.5 (4.3) †		29.2 (4.2) †		29.5 (4.3) †		0.2097
<30 years	5093	55.0	520	10.2	4573	89.8	
≥30 years	4168	45.0	393	9.4	3775	90.6	
**Examined year**							<0.0001
2013	1478	16.0	127	8.6	1351	91.4	
2014	1413	15.3	96	6.8	1317	93.2	
2015	1455	15.7	119	8.2	1336	91.8	
2016	1687	18.2	218	12.9	1469	87.1	
2017	1551	16.7	176	11.3	1375	88.7	
2018	1677	18.1	177	10.6	1500	89.4	

† Values are presented as mean (SE).

**Table 2:** Association of restrictive spirometric pattern according to breastfeeding or duration of breastfeeding.

Variables	OR	95% CI	*p*-Value	*p*-Value for Trend
**Breastfeeding duration**				
**Yes**	0.75	(0.60–0.92)	0.0067	0.0004
1–6	0.86	(0.65–1.14)	0.2918	
7–12	0.79	(0.59–1.06)	0.1195	
13–18	0.82	(0.60–1.14)	0.2358	
19–24	0.74	(0.57–0.96)	0.0257	
25–	0.63	(0.49–0.81)	0.0003	
**None**	1.00			
**Age**				<0.0001
40–49	0.22	(0.13–0.36)	<0.0001	
50–59	0.39	(0.25–0.63)	<0.0001	
60–69	0.51	(0.33–0.79)	0.0028	
70–79	0.74	(0.48–1.14)	0.1671	
80+	1.00			
**BMI**				<0.0001
Underweight	1.76	(1.08–2.85)	0.0224	
Obese	2.00	(1.73–2.31)	<0.0001	
Normal	1.00			
**Smoking status**				0.8768
Ever (less than 100)	1.40	(0.66–2.98)	0.3816	
Ever (more than 100)	0.96	(0.71–1.29)	0.7746	
Never	1.00			
**Asthma**				0.5878
Diagnosed	1.10	(0.78–1.57)	0.5878	
Never diagnosed	1.00			
**Pulmonary tuberculosis**				0.1737
Diagnosed	1.27	(0.90–1.79)	0.1737	
Never diagnosed	1.00			
**Hypertension**				0.0437
Diagnosed	1.19	(1.01–1.40)	0.0437	
Never diagnosed	1.00			
**Diabetes Mellitus**				0.0003
Diagnosed	1.46	(1.19–1.79)	0.0003	
Never diagnosed	1.00			
**Region**				0.4792
Capital	0.95	(0.82–1.10)	0.4792	
Non-Capital	1.00			
**Employment status**				0.7790
Blue-collar worker	1.01	(0.84–1.21)	0.9578	
White-collar worker	0.97	(0.81–1.17)	0.7492	
Unemployed	1.00			
**Education level**				0.2958
Elementary or lower	0.91	(0.68–1.21)	0.5028	
Middle school	1.03	(0.77–1.37)	0.8614	
High school	1.08	(0.86–1.36)	0.5124	
College or higher	1.00			
**House income level**				0.7543
Very high	1.00	(0.79–1.26)	0.9784	
High	1.16	(0.93–1.44)	0.1852	
Low	1.02	(0.83–1.25)	0.8766	
Very low	1.00			
**Parity**				0.0912
Primipara	0.66	(0.41–1.07)	0.0912	
Multipara	1.00			
**Age at menarche**				0.3458
<15 years	1.08	(0.92–1.26)	0.3458	
≥15 years	1.00			
**Age at the first delivery**				0.9210
<25 years	0.99	(0.84–1.17)	0.921	
≥25 years	1.00			
**Age at the last delivery**				0.1757
<30 years	1.11	(0.95–1.30)	0.1757	
≥30 years	1.00			
**Examined year**				0.0002
2013	0.83	(0.65–1.06)	0.1403	
2014	0.63	(0.49–0.83)	0.0007	
2015	0.75	(0.58–0.96)	0.0232	
2016	1.20	(0.96–1.49)	0.1056	
2017	1.06	(0.85–1.33)	0.6027	
2018	1.00			
Values are presented as adjusted odds ratio (95% confidence interval).				

**Table 3:** Coefficients of pulmonary function test results according to breastfeeding or duration of breastfeeding.

FT Results	Variables	Coefficient	*p*-value	*p*-Value for Trend
**FEV1**	**Breastfeeding**			
	**Ever**	0.0390	0.0001	0.0004
	1–6	0.0303	0.0213	
	7–12	0.0421	0.0023	
	13–18	0.0296	0.0498	
	19–24	0.0385	0.0033	
	25–	0.0506	<0.0001	
	**Never**	(reference)		
**FVC**	**Breastfeeding**			
	**Ever**	0.0521	<0.0001	<0.0001
	1–6	0.0407	0.0112	
	7c12	0.0461	0.0062	
	13–18	0.0422	0.0219	
	19–24	0.0521	0.0011	
	25–	0.0732	<0.0001	
	**Never**	(reference)		
**FVC** **percentage (%)**	**Breastfeeding**			
	**Ever**	0.9540	0.0051	0.0002
	1–6	0.7174	0.1007	
	7–12	0.6514	0.1552	
	13–18	0.3390	0.4989	
	19–24	1.0267	0.0181	
	25–	1.6906	<0.0001	
	**Never**	(reference)		
**FEV1/FVC**	**Breastfeeding**			
	**Ever**	−0.0006	0.7038	0.1956
	1–6	−0.0002	0.9264	
	7–12	0.0015	0.4928	
	13–18	−0.0012	0.6094	
	19–24	−0.0007	0.7334	
	25–	−0.0023	0.2588	
	**Never**	(reference)		

## Data Availability

The data are available from the KCDA and Prevention database on the following webpage https://knhanes.kdca.go.kr/knhanes/eng/index.do (accessed on 4 December 2022).

## Supplementary Materials

The following supporting information can be downloaded at: https://www.mdpi.com/article/10.3390/ijerph192316291/s1, Table S1: Demographic characteristics of participants according to Breastfeeding.

## Abbreviations

BMI	body mass index
KNHANES	Korean National Health and Nutrition Examination Survey
KCDA	Korea Disease Control and Prevention Agency
PFT	pulmonary function test
RSP	restrictive spirometric pattern
TLC	total lung capacity
