# Supplementary material for: Association between Breastfeeding and Restrictive Spirometric Pattern in Women Aged over 40 Years: A Cross-Sectional Study
Source: Int J Environ Res Public Health. 2022 Dec 5;19(23):16291. doi: 10.3390/ijerph192316291 (PMC9738453; doi:10.3390/ijerph192316291)
Supplement: Supplementary file 1 [file ijerph-19-16291-s001.zip › ijerph-2049593-supplementary.pdf]

Table S1. Demographic characteristics of participants according to Breastfeeding

Variables	Total (n)		Breastfeeding Ever		Breastfeeding Never		*P-value*
	n	%	n	%	n	%	
	**9261**		**7933**	**85.7**	**1328**	**14.3**	
**Age (years)**	57.5 (10.7)†		58.3 (10.7)†		52.3 (8.6)†		<0.0001
40-49	2519	27.2	1936	76.9	583	23.1	
50-59	2925	31.6	2421	82.8	504	17.2	
60-69	2306	24.9	2136	92.6	170	7.4	
70-79	1369	14.8	1305	95.3	64	4.7	
80+	142	1.5	135	95.1	7	4.9	
**BMI (kg/m2)**	24.0 (3.2)†		24.1 (3.2)†		23.4 (3.3)†		<0.0001
Underweight	189	2.0	143	75.7	46	24.3	
Obese	3127	33.8	2783	89.0	344	11.0	
Normal	5945	64.2	5007	84.2	938	15.8	
**FEV1 (L)**	2.30 (0.45)†		2.28 (0.45)†		2.41 (0.44)†		
**FVC (L)**	2.91 (0.51)†		2.89 (0.51)†		3.02 (0.51)†		
**FVC Percentage (%)**	92.76 (11.58)†		92.76 (11.57)†		92.75 (11.67)†		
**FEV1/FVC**	0.79 (0.06)†		0.79 (0.06)†		0.80 (0.05)†		
**Smoking status**							<0.0001
Ever (less than 100)	76	0.8	65	85.5	11	14.5	
Ever (more than 100)	619	6.7	484	78.2	135	21.8	
Never	8566	92.5	7384	86.2	1182	13.8	
**Asthma**							0.8655
Diagnosed	307	3.3	264	86.0	43	14.0	
Never diagnosed	8954	96.7	7669	85.6	1285	14.4	
**Pulmonary tuberculosis**							0.0487
Diagnosed	326	3.5	267	81.9	59	18.1	
Never diagnosed	8935	96.5	7666	85.8	1269	14.2	
**Hypertension**							<0.0001
Diagnosed	2515	27.2	2294	91.2	221	8.8	
Never diagnosed	6746	72.8	5639	83.6	1107	16.4	
**Diabetes Mellitus**							0.0002
Diagnosed	860	9.3	773	89.9	87	10.1	
Never diagnosed	8401	90.7	7160	85.2	1241	14.8	
**Region**							<0.0001
Capital	4366	47.1	3673	84.1	693	15.9	
Non-Capital	4895	52.9	4260	87.0	635	13.0	
**Employment status**							<0.0001
Blue-collar worker	1863	20.1	1678	90.1	185	9.9	
White-collar worker	2900	31.3	2362	81.4	538	18.6	
Unemployed	4498	48.6	3893	86.5	605	13.5	
**Education level**							<0.0001

Elementary or lower	2863	30.9	2713	94.8	150	5.2	
Middle school	1334	14.4	1231	92.3	103	7.7	
High school	3054	33.0	2462	80.6	592	19.4	
College or higher	2010	21.7	1527	76.0	483	24.0	
**House income level**							<0.0001
Very low	1940	20.9	1779	91.7	161	8.3	
Low	2340	25.3	2045	87.4	295	12.6	
High	2329	25.1	1955	83.9	374	16.1	
Very high	2652	28.6	2154	81.2	498	18.8	
**Parity**							<0.0001
Primipara	347	3.7	238	68.6	109	31.4	
Multipara	8914	96.3	7695	86.3	1219	13.7	
**Age at menarche**	14.6 (1.9)†		14.7 (1.9)†		14.0 (1.8)†		<0.0001
< 15 years	4850	52.4	3948	81.4	902	18.6	
≥ 15 years	4411	47.6	3985	90.3	426	9.7	
**Age at the first delivery**	25.0 (3.9)†		24.7 (3.8)†		26.9 (4.0)†		<0.0001
< 25 years	4541	49.0	4187	92.2	354	7.8	
≥ 25 years	4720	51.0	3746	79.4	974	20.6	
**Age at the last delivery**	29.5 (4.3) †		29.4 (4.3)†		29.8 (4.1)†		0.0002
< 30 years	5093	55.0	4426	86.9	667	13.1	
≥ 30 years	4168	45.0	3507	84.1	661	15.9	
**Examined year**							0.9693
2013	1478	16.0	1266	85.7	212	14.3	
2014	1413	15.3	1214	85.9	199	14.1	
2015	1455	15.7	1250	85.9	205	14.1	
2016	1687	18.2	1435	85.1	252	14.9	
2017	1551	16.7	1325	85.4	226	14.6	
2018	1677	18.1	1443	86.0	234	14.0	

†Values are presented as mean (SE)
